# Pulmonary Nodule Detection Model Based on SVM and CT Image Feature-Level Fusion with Rough Sets

**DOI:** 10.1155/2016/8052436

**Published:** 2016-09-18

**Authors:** Tao Zhou, Huiling Lu, Junjie Zhang, Hongbin Shi

**Affiliations:** ^1^School of Science, Ningxia Medical University, Ningxia, Yinchuan 750004, China; ^2^Department of Urology, The Generel Hospital of Ningxia Medical University, Ningxia, Yinchuan 750004, China

## Abstract

In order to improve the detection accuracy of pulmonary nodules in CT image, considering two problems of pulmonary nodules detection model, including unreasonable feature structure and nontightness of feature representation, a pulmonary nodules detection algorithm is proposed based on SVM and CT image feature-level fusion with rough sets. Firstly, CT images of pulmonary nodule are analyzed, and 42-dimensional feature components are extracted, including six new 3-dimensional features proposed by this paper and others 2-dimensional and 3-dimensional features. Secondly, these features are reduced for five times with rough set based on feature-level fusion. Thirdly, a grid optimization model is used to optimize the kernel function of support vector machine (SVM), which is used as a classifier to identify pulmonary nodules. Finally, lung CT images of 70 patients with pulmonary nodules are collected as the original samples, which are used to verify the effectiveness and stability of the proposed model by four groups' comparative experiments. The experimental results show that the effectiveness and stability of the proposed model based on rough set feature-level fusion are improved in some degrees.

## 1. Introduction

Lung cancer is a malignant tumor with the highest morbidity and mortality rate in the world, posing a serious threat to human life and health [1, 2]. The ability to estimate the risk of lung cancer is important in two common clinical models [3]: pulmonary nodules management and risk prediction model. Identification of early symptomatic in lung cancer is very important to improve early survival and reduce emergency presentations. Early detection is the most popular method to improve the effectiveness of the treatment of patients with lung cancer. Since pulmonary nodules are the early form of lung cancer [4], the detection of pulmonary nodules plays a critical role in the early diagnosis and treatment of lung cancer. Recent advances in computed tomography (CT) have a progressively increased spatial resolution and decreased acquisition times, making it possible for high resolution, multiangle, 3-dimensional, isotropic image of the whole lung to be acquired in less than 10 seconds. This has expanded capabilities for the early detection of small pulmonary nodules [4]. It is believed that early detection of lung cancer will result in earlier treatment at lower stages of the disease, thereby improving the 5-year survival rate, which has remained relatively constant at 15% for the last 30 years. However, with the wide application of CT in the lung imaging, the issues of CT data overloading and subjective interpretation of images result in a high clinical misdiagnosis rate [5].

Computer-Aided Diagnosis (CAD) systems provided a beneficial support and enhance the diagnostic accuracy. CAD is capable of performing the preliminary screen of the vast amounts of CT image and marking suspicious lesions, thereby helping radiologists to carry out the quadratic discrimination to reduce the workload and improve the accuracy rate of cancer diagnosis [6, 7].

Pulmonary nodule detection technology is one of the hot topics in the field of CAD in recent years. For example, ROI segment is a key problem, Xia et al. [8] using local variational Gaussian mixture models to segment brain MRI image Based on Learning Local Variational Gaussian Mixture Models, segmentation of breast ultrasound images are discussed by Xian [9, 10] and Santos et al. [11] segment the lung parenchyma based on region growing algorithm. Magalhães Barros Netto et al. [12] use growing neural gas (GNG) to segment the lung parenchyma, the obtained pulmonary nodules are then separated from tissues containing blood vessels and bronchi according to the 3D distance transform, and finally SVM is used to carry out the effective identification of pulmonary nodules with shape and texture features. Ye et al. [13] firstly segment and extract region of interest (ROI) with fuzzy threshold in combination with Gaussian matrix, mean curvature, and Hessian matrix, then choose the local shape information and local intensity dispersion as the feature expression of ROI, and finally use the weighted SVM for recognition of pulmonary nodules. Tan et al. [14] segment pulmonary nodules based on the blood vessels and nodule enhancement filter proposed by Li et al. [15], then locate the clustering center of pulmonary nodules based on the divergence calculated by Gaussian template and achieve ROI extraction, and finally use the classifier based on genetic model, artificial neural network (ANN), and SVM for comparative analysis of the detection effectiveness of pulmonary nodules; Cascio et al. [16] use regional growth model and morphological operation to extract the ROI firstly, then reconstruct B-spline surface based on 3D spring model in order to extract the related 3D gray features and shape features, and detect the pulmonary nodules using ANN. Although the above literature explores the methods of detecting pulmonary nodules, overall, these are still two disadvantages of these methods in feature structure design and feature set expression as follows.

1. When extracting and quantifying feature for ROI, the feature structure design is irrational, reflected by the fact that the combination of global features and local features and the combination of two-dimensional and three-dimensional features are not fully considered.
2. When fusing feature data, the compactness of feature expression is a difficult problem. Therefore, feature redundancy is usually not eliminated. Moreover, the feature-level fusion method without prior knowledge is rarely used.

Rough set theory was developed by Zdzislaw Pawlak in the early 1980s and can be regarded as a new mathematical tool for feature selection, feature extraction, and decision rule generation without prior knowledge. Rough sets provide the mechanism to find the minimal set of attributes required to classify the training samples. This minimal set of attributes is called reduct and contains the same knowledge as the original set of attributes in a given information system. Therefore, reducts can be used to obtain different classifiers. Wang et al. [17] present a framework for a systematic study of the rough set theory. Various views and interpretations of the theory and different approaches to study the theory are discussed. The relationships between the rough sets and other theories, such as fuzzy sets, evidence theory, granular computing, formal concept analysis, and knowledge spaces, are examined. Cost of disease prediction and diagnosis can be reduced by applying machine learning and data mining methods. Disease prediction and decision-making play a significant role in medical diagnosis. Udhaya Kumar and Hannah Inbarani [18] put forward a novel neighborhood rough set classification approach to deal with medical datasets. Experimental result of the proposed classification algorithm is compared with other existing approaches such as rough set, *K*th-nearest neighbor, support vector machine, BP NN, and multilayer perceptron to conclude that the proposed approach is a cheaper way for disease prediction and decision-making. Feature Selection (FS) is a solution that involves finding a subset of prominent features to improve predictive accuracy and to remove the redundant features. Thus, the learning model receives a concise structure without forfeiting the predictive accuracy built by using only the selected prominent features. Therefore, nowadays, FS is an essential part of knowledge discovery. Inbarani et al. [19] proposed new supervised feature selection methods based on hybridization of Particle Swarm Optimization (PSO), PSO based Relative Reduct (PSO-RR), and PSO based Quick Reduct (PSO-QR) presented for the diseases diagnosis, in order to seek to investigate the utility of a computer-aided diagnosis in the task of differentiating malignant nodules from benign nodules based on single thin-section CT image data. In Shah et al. [20], CT images of solitary pulmonary nodules were contoured manually on a single representative slice by a thoracic radiologist. Two separate contours were created for each nodule, one including only the solid portion of the nodule and one including any ground-glass components. For each contour, 75 features were calculated that measured the attenuation, shape, and texture of the nodule. These features were then input into a feature selection step and four different classifiers to determine if the diagnosis could be predicted from the feature vector. Hassanien [21] discuss a hybrid scheme that combines the advantages of fuzzy sets and rough sets in conjunction with statistical feature extraction techniques. An application of breast cancer imaging has been chosen and hybridization scheme have been applied to see their ability and accuracy to classify the breast cancer images into two outcomes: cancer or noncancer.

Based on the above reasons, a pulmonary nodule detection model based on rough set (RS) feature-level fusion and SVM is proposed in this paper. To overcome the first aforementioned disadvantage, the shape feature, intensity feature and texture feature are extracted. For shape feature, three new 3-dimensional features, namely, External Spherical Volume (ESV), Surface-Center Distance Standard Deviation (SCDSTD), and External Rectangle Cross Line Distance (ERCLD) are proposed. For intensity feature, three new 3-dimensional features, namely intensity gradient (from inside to outside), Laplace Divergence Mean (LDM), and Laplace Divergence Distance (LDD) are proposed. Regarding feature description, two-dimensional texture feature, three-dimensional shape feature, and intensity feature are used for quantification. With regard to the second aforementioned disadvantage, rough set feature-level fusion is adopted since it can fully retain the properties of the features without prior knowledge. Finally, a grid optimization model is employed to optimize the kernel function of support vector machine (SVM), which is used to conduct the recognition and detection of pulmonary nodules. In order to verify the validity and stability, advantages of the model, four groups of comparative experiments are performed in this paper, that is, model validation experiments before and after rough set reduction, model stability experiments before and after rough set reduction, validation experiments of the superiority of the rough set feature-level fusion model, and comparative experiments with other pulmonary nodule detection models to compare the performance. The experimental results show that the method proposed in this paper can improve, to a certain extent, the rationality of feature structure and compactness of feature expression, thereby improving the detection accuracy of pulmonary nodules.

## 2. Related Theory

The description of ROI features is determined by both its comprehensiveness (features cannot be “observed” with “multiperspective” approach if the features amount is too little) and the accuracy of characterization (more quantized values diverged from the real information will cause a low feature discrimination). A large number of noise information sets will reduce the ROI feature extraction accuracy and affect the final results of detection. Therefore, for comprehensive and accurate expression of the morphological structure of ROI and local features, six new 3-dimensional features are proposed based on the analysis of ROI for lung CT image. These new 3-dimensional features are used to qualitatively analyze and quantitatively characterize the lesions from 2-dimensional and 3-dimensional perspectives in combination with other shape features, intensity features, texture features.

### 2.1. Pulmonary Nodules Features in CT Image

#### 2.1.1. Shape Characteristics

Shape characteristics analyze the spatial distribution of gray values, by computing local features at each point in the image. Shape feature is the most intuitive visual feature, which can be used to describe the main medical signs of CT image of pulmonary nodule ROI, such as nodule sign, lobulation sign, spinous process sign, vacuole sign, and spicule sign, from the perspectives of geometric shape, edge roughness, and topology structure. In this paper the extracted components of the shape features mainly include perimeter, area, volume, roundness, rectangularity, elongation, Euler number, Harris, Hu moment, ESV, SCDSTD, and ERCLD. Here some features are given [22]:

*(1) Area*

(1) $$\begin{array}{c} S=\overset{N}{\underset{x=1}{\sum }}\overset{M}{\underset{y=1}{\sum }}f\left(\;x,y\;\right), \end{array}$$

where *f*(*x*, *y*) is the pixels of the target and *M* and *N* are the length and width, respectively.

*(2) Perimeter*

(2) $$\begin{array}{c} C=\overset{M}{\underset{i=1}{\sum }}\overset{N}{\underset{j=1}{\sum }}p\left(\;i,j\;\right), \end{array}$$

where *p*(*i*, *j*) is the pixels of the target edge and *M* and *N* are the length and width, respectively.

*(3) Circularity*

(3) $$\begin{array}{c} R_{0}=\frac{C^{2}}{4\pi S}. \end{array}$$

Circularity describes object shape that is close to the degree of circular, where *S* is the area of the target region and *C* is circumference of the target region. 0 < *R*_0_ < 1 and *R*_0_ value reflects the complexity of the measurement boundary; the shape is more complex and the *R*_0_ value is more smaller.

*(4) Rectangularity*

(4) $$\begin{array}{c} R=\frac{S}{\left(H*W\right)}, \end{array}$$

where *S* is the area of the target region and *H* and, *W* are the length and width, respectively.

*(5) Elongation*

(5) $$\begin{array}{c} E=\frac{\min\left(H,W\right)}{\max\left(H,W\right)}. \end{array}$$

Elongation can distinguish different shapes of the images (such as circle, square, ellipse, thin and long, and short and wide), where *H* and *W* are the length and width, respectively.

*(6) Euler Number*

(6) $$\begin{array}{c} E=C-H, \end{array}$$

where *C* is the number of connection parts and *H* is the number of holes.

**Figure 1:**
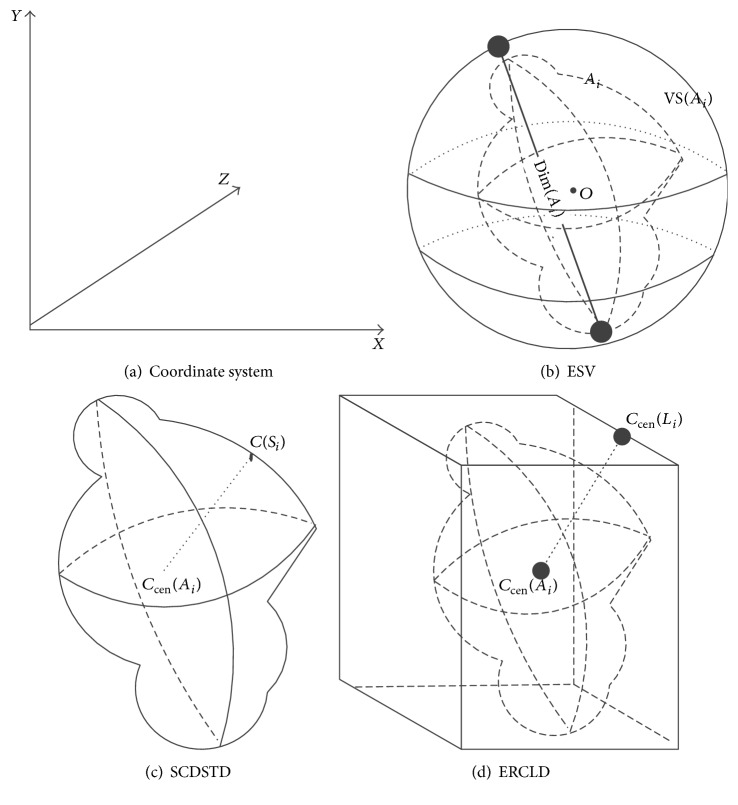
Three-dimensional character sketch.

*(7) External Spherical Volume (ESV).* ESV is the ratio of each ROI *A*_*i*_ (maximum diameter is dim(*A*_*i*_)) to the External Spherical Volume VS(*A*_*i*_) extracted from three-dimensional CT image, which reflects the similarity between the region and the sphere, as shown in Figure 1(b).

(7) VolumeVSAi=43×π×dim23E1Ai=VolumeAiVolumeVSAi.

*(8) Surface-Center Distance STandard Deviation (SCDSTD).* SCDSTD is the coordinate distance standard deviation of each individual element *C*(*S*_*i*_) and regional center *C*_cen_(*A*_*i*_) from the surface of each ROI; its value also describes the similarity with sphere of ROI. If the value is 0, *E*_2_(*A*_*i*_) is a standard sphere. With the increase in *E*_2_(*A*_*i*_) value, the magnitude of the deviation from the sphere in the region increases, as shown in Figure 1(c).

(8) $$\begin{array}{c} E_{2}\left(\;A_{i}\;\right)=\text{std}\left(\;\frac{\left\|C\left(S_{i}\right)-C_{cen}\left(A_{i}\right)\right\|}{mean\left(\left\|C\left(S_{i}\right)-C_{cen}\left(A_{i}\right)\right\|\right)}\;\right). \end{array}$$

*(9) External Rectangle Cross Line Distance (ERCLD).* ERCLD is the distance from center voxel *C*_cen_(*A*_*i*_) of ROI to the center dim⁡(*L*_*i*_)  (*i* = 1,2,…, 12) of its 12 intersecting lines, which may indicate that the regional voxel is evenly distributed in the rectangular body, as shown in Figure 1(d).

(9) $$\begin{array}{c} E_{3}\left(\;A\;\right)=\frac{\left\|\text{mean}\left(C_{cen}\left(A_{i}\right)-C_{cen}\left(dim\left(L_{i}\right)\right)\right)\right\|}{mean\left(C_{cen}\left(dim\left(L_{i}\right)\right)\right)}. \end{array}$$

#### 2.1.2. Hu Moment Characteristics

Moments and the related invariants have been extensively analyzed to characterize the patterns in images. The moment invariants are independent of position, size, and orientation but also independent of parallel projection. Hu [23] was the first person to prove the central moment invariants. The central geometric moment invariants are derived based upon algebraic invariants, including six absolute orthogonal invariants and one skew orthogonal invariant. The moment invariants have been proved to be the adequate measures for tracing image patterns about the images translation, scaling, and rotation.

Hu moment invariants define seven values, computed by normalizing central moments through order three, which are invariant to object scale, position, and orientation, and a large number of papers that have significant contribution to the application of Hu moment. Two-dimensional moments of a digitally sampled *M∗N* image that has gray function *f*(*x*, *y*)  (*x* = 1,2,…, *M*, *y* = 1,2,…, *N*) are given as

(10) $$\begin{array}{c} M_{p,q}=\overset{M}{\underset{x=1}{\sum }}\overset{N}{\underset{y=1}{\sum }}x^{p}y^{q}f\left(\;x,y\;\right)\;p,q=1,2,3,\ldots . \end{array}$$

The moments *f*(*x*, *y*) translated by an amount (*a*, *b*) are defined as

(11) x¯=m10m00,y¯=m01m00μp,q=∑x=1M∑y=1Nx−x¯py−y¯qfx,yp,q=1,2,3,….

When a scaling normalization is applied, the central moments change as

(12) $$\begin{array}{c} \eta_{p,q}=\frac{\mu_{p,q}}{\mu_{00}^{\gamma }},\;\gamma =\left(\frac{\left(p+q\right)}{2}\right)+1. \end{array}$$

In terms of the central moments, the seven moments are given as

(13) C1=η20+η02C2=η20−η022+4η112C3=η30−3η122+3η21−η032C4=η30+η122+η03+η212C5=η30−3η12η30+η12·η30+η122−3η03+η212+3η21−η03·η21+η033η30+η122−η03+η212C6=η20−η02η30+η122−η03+η212+4η11η30+η12η21+η03C7=3η21−η03η30+η12·η30+η122−3η03+η212+3η12−η30·η21+η033η30+η122−η03+η212.

Hu 7-moment invariants vary widely, in order to compare, using logarithmic function to compress data, and hence the actual invariants moment features are *C*_*K*_′:

(14) $$\begin{array}{c} C_{K}=\left|\;l{\mathrm{og}}_{10}\left|\;C_{K}^{\prime }\;\right|\;\right|\;K=1,2,\ldots ,7. \end{array}$$

The amended moment invariant features possess translation invariance, rotational invariance, and scale invariance.

#### 2.1.3. Texture Characteristics

Tamura texture features, Tamura texture based on human visual perception in psychological research, are proposed by Tamura in 1978. Six components of Tamura texture feature correspond with 6 properties in psychology, three of them are coarseness, contrast, and directionality, which have the good application value in the texture synthesis, image recognition, and so on.

Texture is the gray distribution which appears repeatedly in the space position, so there are some relationships between two pixels at some distance from each other in image space, called gray spatial correlation properties in gray image. GLCM is a common method by studying the relevant relationship of gray image.

#### 2.1.4. Intensity Features

Gray statistical feature is a quantitative method to describe the basic features of two-dimensional image region; it is called intensity feature from three-dimensional perspective [16]. In this paper, the extracted components of intensity features include the mean intensity, intensity variance, maximum and minimum intensity difference, skewness, kurtosis, intensity gradient (from inside to outside), Laplace Divergence Mean (LDM), and Laplace Divergence Distance (LDD).

*(1) Intensity Gradient (from Inside to Outside).* For ROI *A*_*i*_ with the voxel *S*_*i*_ volume greater than 0, morphological erosion processing is performed continuously and the ratio of the mean of the excluded area of each erosion processing to the mean of the last operation (initial value is 0) is calculated until the ratio is zero. Consider the following equation where *n* is the number of operations.

(15) $$\begin{array}{c} E_{4}\left(\;A_{i}\;\right)=\frac{K}{n}. \end{array}$$

*(2) Laplace Divergence Mean (LDM).* According to the Laplacian convolution results with the original CT image, it is found that the nodule surrounding area with smaller gray value difference has a significant different divergence. Therefore, calculation of Laplace divergence is helpful to distinguish pulmonary nodules from interfering impurities.

(16) $$\begin{array}{c} E_{5}\left(\;A_{i}\;\right)=\text{mean}\left(\;A_{i}\times La\;\right). \end{array}$$

*(3) Laplace Divergence Distance (LDD).* The difference between the maximum and minimum values of the Laplace divergence values is used to describe the range of regional divergence.

(17) $$\begin{array}{c} E_{6}\left(\;A_{i}\;\right)=\max\left(\;A_{i}\times La\;\right)-\min\left(\;A_{i}\times La\;\right). \end{array}$$

Table 1 shows the feature set of 42 features based on the above feature description of ROI. To facilitate subsequent tests, features are numbered in the order as showed in Table 1; that is, the shape features are numbered fs1–fs18, the intensity features are numbered fi1–fi8, and texture features are numbered ft1–ft16, respectively.

### 2.2. Rough Set and Attribute Reduction

Rough set theory (RST), proposed by Pawlak in 1982, is one of the effective mathematical tools for processing fuzzy and uncertainty knowledge. Nowadays, RST has been applied to a variety of fields such as artificial intelligence, data mining, pattern recognition, and knowledge discovery. Rough set is founded on the assumption that with every object of the universe of discourse some knowledge is associated. Objects characterized by the same information are similar in view of the available information about them. The indiscernibility relation generated in this way is the mathematical basis of rough set theory. Any set of all indiscernible objects are called an elementary set and form a basic granule of knowledge about the universe. Any union of some elementary sets is referred to as a crisp set, otherwise the set is rough set.

**Definition 1 .**

An information system *S* is a quadruple *S* = (*U*, *A*, *V*, *f*), where *U* is a nonempty and finite set of objects, *A* is a nonempty and finite set of attributes, *V*≔⋃*V*_*a*_ with *V*_*a*_ being the domain of attribute *a*, and *f* is an information function such that *f*(*x*, *a*) ∈ *V*_*a*_ for every *x* ∈ *U* and every *a* ∈ *A*. A decision system is an information system (*U*, *C* ∪ *D*, *V*, *f*) with *C*∩*D* = *Ф*, where *C* and *D* are called the conditional and decision attribute sets, respectively.

For a subset *P* of *A*, let us define the corresponding equivalence relation as

(18) INDP=x,y∈U×U ∣ fx,a=fy,a  for  any  a∈P

and denote the equivalence class of IND(*P*) which contains the object *x* ∈ *U* by [*x*]_*P*_; that is,

(19) $$\begin{array}{c} {\left[\;x\;\right]}_{P}=\left\{\;y\in U\;|\;\left(\;x,y\;\right)\in \text{IND}\left(\;P\;\right)\;\right\}. \end{array}$$

The factor set of all equivalence classes of IND(*P*) is denoted by *U*/*P*; that is, *U*/*P* = {[*x*]_*P*_∣*x* ∈ *U*}.

As well known, attribute reduction is one of the key issues in RST. It is performed in information systems by means of the notion of a reduct based on a specialization of the notion of independence due to Marczewski. Up to now, much attention has been paid to this issue and many different methods of attribute reduction have been proposed for decision systems. For example, the reduction approaches are, respectively, based on partition, discernibility matrix, conditional information entropy, positive region, and ant colony optimization approach.

**Definition 2 .**

Let *S* = (*U*, *A*, *V*, *f*) be an information system and *P*⊆*A*. For a subset *X* of *U*, *R*_*P*_(*X*) = {*x* ∈ *U*∣[*x*]_*P*_⊆*X*} and *R* ^*P*^(*X*) = {*x* ∈ *U*∣[*x*]_*P*_∩*X* ≠ *Ф*} are called *P*-lower and *P*-upper approximations of *X*, respectively.

**Definition 3 .**

Let *S* = (*U*, *A*, *V*, *f*) be an information system and let *P* and *Q* be two subsets of *A*. Then, POS_*P*_(*Q*) = ⋃_*X*∈*U*/*Q*_ *R*_*P*_(*X*) is called *P*-positive region of *Q*, where *R*_*P*_(*X*) is the *P*-lower approximation of *X*.

**Definition 4 .**

Let *S* = (*U*, *A*, *V*, *f*) be a decision system, *a* ∈ *C*, and *P*⊆*C*. If POS_*C*_(*Q*) = POS_*C*∖{*a*}_(*Q*), *a* is said to be *D*-dispensable in *C*; otherwise, *a* is said to be *D*-indispensable in *C*. The set of all the *D*-indispensable attributes is called the core of *S* and denoted by Core(*S*). Furthermore, if POS_*P*_(*Q*) = POS_*C*_(*Q*) and each of the attributes of *P* is *D*-indispensable, then *P* is called a reduct of *S*.

### 2.3. SVM and Its Optimization

SVM is a pattern recognition method developed from statistical learning theory based on the idea of structural risk minimization principle. In the case of ensuring classification accuracy, SVM can improve the generalization ability of the learning machine by maximizing the classification interval. The biggest advantage of SVM is that it overcomes the overlearning and high dimension both of which lead to computational complexity and local extremum problems. A reliable classification model based on SVM is urgently needed for the study of hospitalization expenses of patients with gastric cancer.

**Figure 2:**
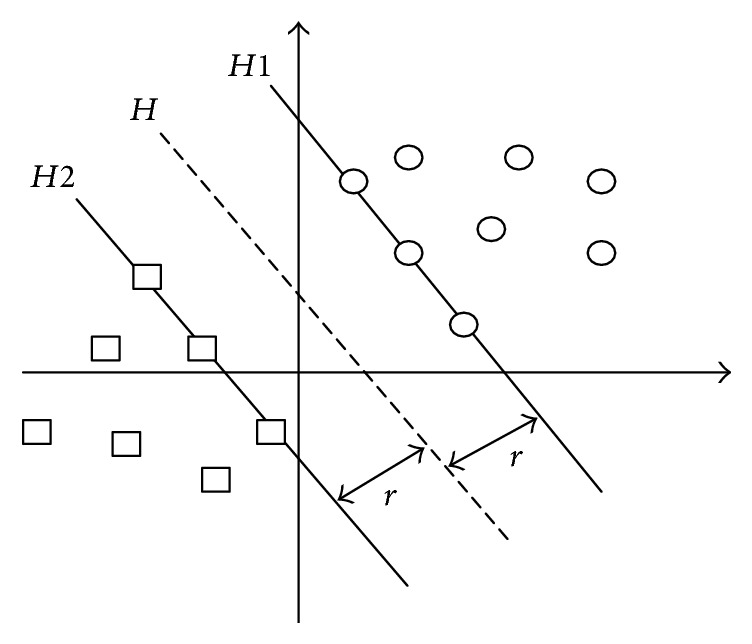
Optimal hyper plane.

SVM deals with linearly separable data (Figure 2); the assumption is that there are data sets *S* = {*x*_1_,…, *x*_*n*_} and data marker *G* = {*y*_1_,…, *y*_*n*_}, where *x*_*i*_ is the input space vector of the data sample and *y*_*i*_ records the category of the sample.

The aim of SVM is to find an optimal hyper plane *H* to separate these two samples and make the largest interval. The optimal hyper plane *H* is expressed as

(20) $$\begin{array}{c} w^{T}x+b=0, \end{array}$$

where *w* is the weight vector and *b* is the threshold.

This problem is transformed into the optimal problem of *w* and *b*:

(21) minw,b rw=12w yiw·x+b≥1,i=1,…,n.

In order to simplify the formula, the Lagrange dual is introduced to meet the requirements of KKT (Karush-Kuhn-Tucker). The objective function is transformed into

(22) minα 12∑i=1n∑j=1nyiyjαiαjxi·xj−∑j=1nαjs.t. ∑i=1nyiαi=0,αi≥0,  i=1,2,…,n.

As for the linearly inseparable data, the penalty parameter *C* and relaxation variable *ξ* are introduced in the constraint condition, thus the generalization ability of SVM is increased, and the function is transformed into

(23) minα 12∑i=1n∑j=1nyiyjαiαjxi·xj−∑j=1nαjs.t. ∑i=1nyiαi=0,0≤αi≤C,

where *C* is the artificial setting parameter. According to the practical experience, the bigger *C*, the greater separation interval. At the same time, it will increase the risk of generalization.

The final classification function is

(24) $$\begin{array}{c} f\left(\;x\;\right)=\mathrm{sgn}\left\{\;\left(\;\overset{n}{\underset{i=1}{\sum }}a_{i}^{*}y_{i}\left(\;x_{i}\cdot x\;\right)\;\right)+b^{*}\;\right\}. \end{array}$$

For nonlinear classification data, SVM transforms them into linearly separable data in a high-dimensional space via nonlinear mapping of kernel function, and the optimal hyper plane is found in high-dimensional space. The kernel function which meets the mercer kernel condition corresponding to the transvection of a spatial transformation is used to realize the nonlinear transformation of linear classification.

The corresponding kernel function is defined as

(25) $$\begin{array}{c} K\left(\;x_{i},x\;\right)=\left(\;\varphi \left(\;x_{i}\;\right),\varphi \left(\;x\;\right)\;\right). \end{array}$$

At this point the final classification function is

(26) $$\begin{array}{c} f\left(\;x\;\right)=\mathrm{sgn}\left\{\;\left(\;\overset{n}{\underset{i=1}{\sum }}a_{i}^{}y_{i}K\left(\;x_{i}\cdot x\;\right)\;\right)+b\;\right\}. \end{array}$$

Penalty factor *C* and parameter *g* of the kernel function play an extremely important role in the performance of SVM classification. In order to obtain the optimal classification results, grid optimization model is used for optimization in this paper. In grid optimization model, the parameters to be searched are expressed in the form of grids in a certain space, and the optimal parameters are selected by traversing all the grids. Therefore, grid optimization model has the advantages of simplicity, convenience, good stability, and easiness to get the global optimal solution [24]. In the learning process of SVM, 10-fold cross-validation is used to calculate the kernel function parameters and penalty coefficient with the optimal classification performance, which are then applied to the SVM classifier for recognition and detection of pulmonary nodules. Finally, sensitivity, specificity, accuracy, and processing time are used as indexes to evaluate the detection of relevant experiments.

## 3. Pulmonary Nodule Detection Model

**Figure 3:**
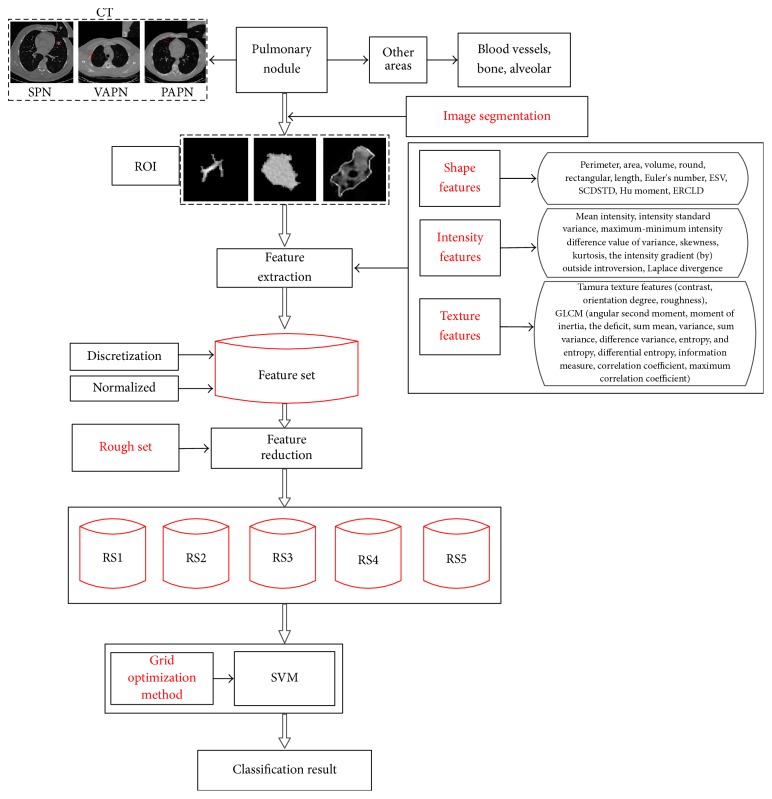
Flow chart of pulmonary nodule detection model.

In this paper, CT images of 70 cases of patients with pulmonary nodules are used. The images are firstly segmented [7] to three different types of pulmonary nodules (solitary pulmonary nodules or SPN, vascular adhesion pulmonary nodules or VAPN, and pleural adhesion pulmonary nodules or PAPN), which are marked by radiologists, as well as a large number of nonnodular areas, including blood vessels, bones, and alveoli. Forty-two feature components characterizing ROI are extracted from the 2-dimensional and 3-dimensional perspectives, including six new 3-dimensional features proposed in this paper. They are composed of 18 shape features, 8 intensity features, and 16 texture features. The extracted feature set (identified as the FS) is discretized and normalized. Feature-level fusion of the improved feature data is performed for five times using rough set model (since the reduction of rough set feature subset is not unique, in this paper, the extracted feature sets are reduced for five times and are identified as RS1, RS2, RS3, RS4, and RS5). Feature subset RS1 is used for comparative experiment. Finally, SVM parameters are optimized using grid optimization model, and the improved SVM is used in the following four sets of comparative experiments: comparative analysis of the effectiveness and stability of classification before and after rough set reduction of features; comparative analysis of the recognition performance before and after feature-level fusion based on rough set or PCA; comparative analysis of the recognition performance of our proposed method and other methods. Based on the above views, we present a flow chart of pulmonary nodule detection model as shown in Figure 3.

## 4. Results and Discussion

### 4.1. Experimental Environments

In this paper, the hardware and software environments are as follows.

*Software Environments.* Windows 7 OS, the Matlab R2014b, ImageJ 1.48 u, and LibSVM.

*Hardware Environments.* Intel Core i5 4670-3.4 GHz, 8.0 GB of memory, and 500 GB hard disk.

**Figure 4:**
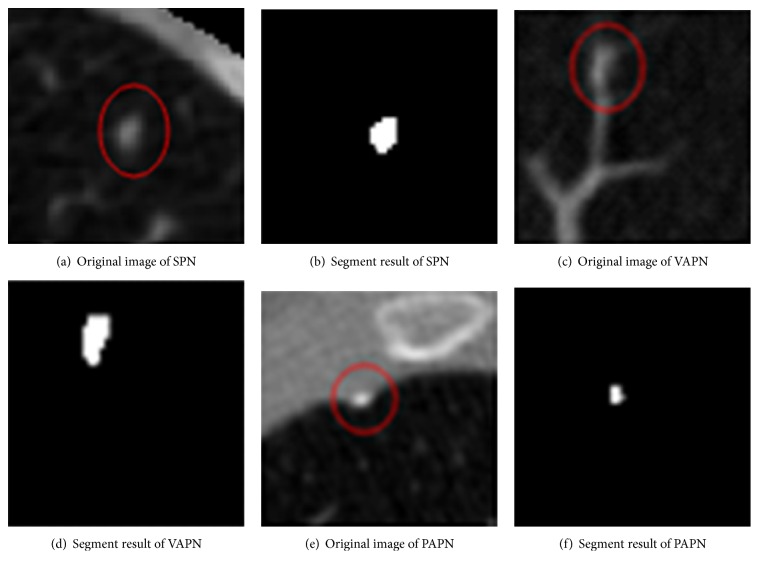
Pulmonary nodule segmentation results.

*Experimental Data.* CT images of 70 cases of patients with pulmonary nodules are collected as experimental samples, which are marked by radiologists, with a size of 512 × 512 and a thickness of 2 mm. They are composed of 2232 CT images from 38 cases of patients with solitary pulmonary nodules (SPN), 17 cases of patients with vascular adhesion pulmonary nodules (VAPN), and 15 cases of patients with pleural adhesion pulmonary nodules (PAPN), respectively.  Figure 4 shows the representatives of each type of pulmonary nodules and the corresponding segmentation results.

**Figure 5:**
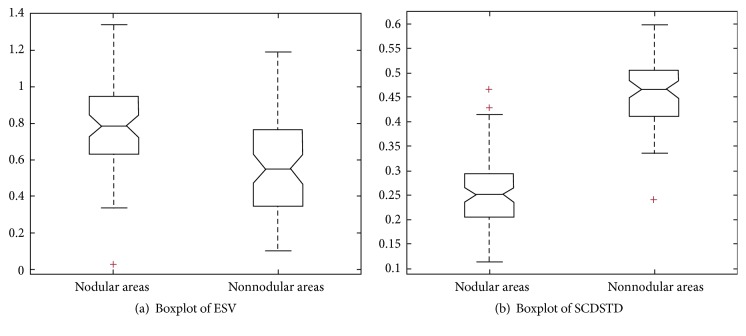
Pulmonary nodule area and the pulmonary nodules boxplot. “+” refers to upper and lower bounders of ESV value and SCDSTD value.

 In this paper, 42-dimensional features of 70 marked pulmonary nodular areas and 70 randomly selected nonnodular areas are extracted.  Table 2 shows the 42-dimensional feature values of the lung nodular and nonnodular areas. shape features are identified as the fs, intensity features are identified as the fi, and texture features are identified as the ft. In order to intuitively understand the distribution of different feature values and the discrimination comparison, external sphere volume (ESV) ratio and the standard deviation of surface-center distance (SCD) are calculated and plotted as box diagram as shown in Figure 5.

### 4.2. Feature-Level Fusion Based on Rough Set

In order to avoid the attribute value of small range of values dominated by that of large range of values and reduce the complexity of the statistical computation process, the extracted feature sets are firstly preprocessed by normalizing data with bigger difference and linearly mapping the data to [0, 1]. The preprocessed feature data are then fused for five times using rough set model. The fusion results are shown in Table 3.

### 4.3. Pulmonary Nodule Detection with SVM Based on Grid Optimization

#### 4.3.1. The Model Effectiveness Experiment

Tenfold cross-validation is used to calculate the accuracy, sensitivity, specificity, and processing time of classification before and after rough set reduction (RS1(70 × 21) obtained from experiment one is used as the data set after reduction), and the recognition performance of classifier is compared before and after reduction. The results are shown in Table 4.

Experimental results show that pulmonary nodule detection accuracy is increased significantly after feature-level fusion, with a decrease in the missed diagnosis rate, reflected by the increased sensitivity, and the misdiagnosis rate, reflected by the increased specificity. The processing time is also shorter after reduction. These results indicate that the feature-level fusion of the extracted feature set with 42 dimensionalities based on rough set model is effective, which not only improves the compactness of the feature set (to eliminate redundancy and low degree of differentiation features component), but also corrects the abnormal data of the feature set, thereby further improving the performance of pulmonary nodule detection.  Table 5 shows the effectiveness of the five rough set reduction subsets.

#### 4.3.2. The Model Stability Experiment

The feature data of pulmonary nodules are tested with RS1(70*∗*21) as the dataset for classification for five rounds with a different ratio of training set over testing set of 50/20, 40/30, 35/35, 35/35, or 20/50. Each round of test is carried out with a randomly selected ratio of training set over testing set and the mean of 10 test results is used as the corresponding accuracy, sensitivity, specificity, and running time of the model. The results are shown in Table 6.

**Figure 6:**
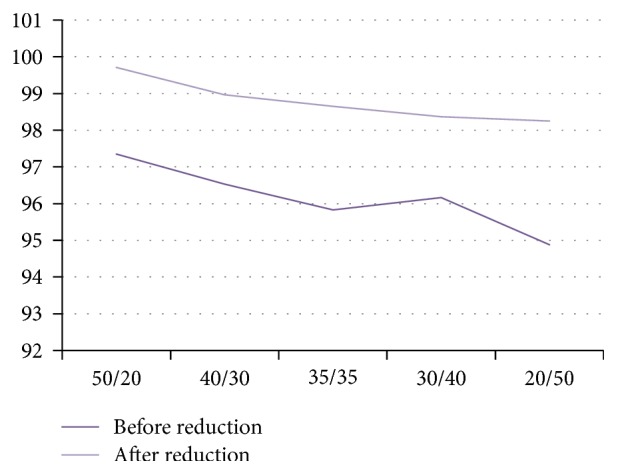
Comparative results of feature subsets before and after rough set reduction.

The experimental results show that, with the decrease in the ratio of training set over testing set, the decrease in the classification accuracy of feature subset after rough set reduction is not obvious, whereas that of feature set before rough set reduction is fluctuating to certain extent (Figure 6 is more intuitive). These results indicate that the classification stability of the feature level fusion model based on rough set is higher and is less susceptible to the interference of sample data.  Table 7 shows the stability of 5 groups feature subset after rough set reduction.

#### 4.3.3. The Superiority of Feature-Level Fusion Model Based on Rough Set

**Figure 7:**
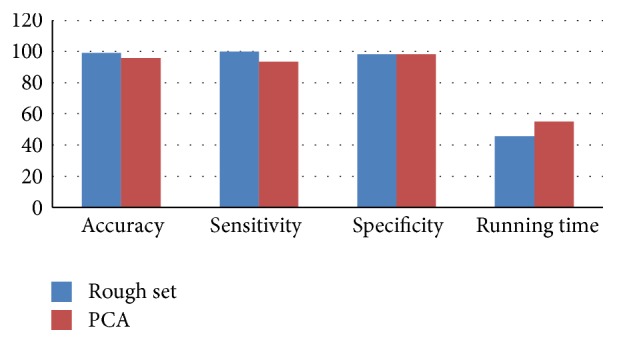
Comparison of two feature-level fusion models.

Since PCA is a well-developed model, characterized by simple calculation and easy programming, it has become the preferred dimension reduction method for most of the feature-level fusion model in order to analyze comparatively two types of feature-level fusions. In this paper, PCA-based feature-level fusion of the extracted feature sets is performed at the same time, and the tenfold cross-validation results are shown in Table 8.  Figure 7 shows the classification performance of the two types of feature-level fusion methods (feature subset RS1 from Table 3 is used, and the running time is 100 × actual time).

Experimental results show that various performance indicators of the feature-level fusion model based on rough set are better than those based on PCA, indicating that the rough set is more suitable than PCA to eliminate redundant information.

#### 4.3.4. Comparison with Other Pulmonary Nodule Detection Methods

Pulmonary nodule detection accuracy and False Positives per scan (FP/s) are used as the evaluation indexes of pulmonary nodule detection methods to compare and analyze the method proposed in this paper and other five detection methods of pulmonary nodules (the optimal detection accuracy is used for all detection methods). The results are shown in Table 9 (Pr: private database; L: LIDC).

Experimental results show that the proposed method is superior to the other pulmonary nodule detection methods to a certain extent, indicating that this method not only improves the comprehensiveness and accuracy of the feature description of ROI by supplementing and improving the feature components, but also improves the firmness of the feature set by integrating the concept of feature-level fusion based on rough set to exclude the redundant features and data with irregular information, thereby improving the overall pulmonary nodule detection performance.

## 5. Conclusions

In this paper the research status quo of pulmonary nodule detection methods is analyzed and a pulmonary nodule detection model is proposed based on rough set based feature-level fusion. To address the issues that the feature description is insufficient and the characterization is inaccurate in the process of feature extraction, six new 3D features, in combination with other 2D and 3D features, are proposed to extract and quantify the feature information of ROI in this model. A rough set based feature-level fusion is employed to reduce the dimensionality of the feature sets since there is redundant information in the extracted high-dimensional features. In addition, a grid optimization model is adopted to optimize the SVM kernel function, which is used as the classifier for detection and recognition of pulmonary nodule. Finally, the pulmonary nodule detection performance of the proposed method is verified with four groups of comparative experiments. The experimental results show that the proposed pulmonary nodule detection method based on rough set based feature-level fusion is effective, with the classification accuracy that can basically meet the requirements of medical imaging for the detection of pulmonary nodules and therefore is of great value for the detection of pulmonary nodules and auxiliary diagnosis of lung cancer.

## Figures and Tables

**Table 1:** ROI feature set.

Feature type	Feature vectors	Dimensionality
Shape features (fs)	Perimeter, area, volume, roundness, rectangularity, length, Euler's number, ESV, SCDSTD, ERCLD, Hu moment	18

Intensity features (fi)	Mean intensity, intensity standard variance, maximum-minimum intensity difference value of variance, skewness, kurtosis, intensity gradient (from inside to outside), LDM, LDD	8

Texture features (ft)	Tamura texture features (contrast, direction, roughness), GLCM (angular second moment, moment of inertia, torque deficit, sum mean, variance, sum variance, difference variance, entropy, sum entropy, differential entropy, information measure, correlation coefficient, maximum correlation coefficient)	16

**Table 2:** Feature values of pulmonary nodular areas and nonnodular areas.

Shape features (fs)		Intensity features (fi)		Texture features (ft)	
Nodular areas	Nonnodular areas	Nodular areas	Nonnodular areas	Nodular areas	Nonnodular areas
95	78	59.06	91.0987	8.3104	5.4016
159	128	14.06	4.4872	12.041	12.5216
284	178	0.5956	−0.39568	0.4303	0.0067
0.6517	0.211	2.7348	1.8669	0.7709	0.7275
0.6961	2.1587	55.1865	14.3481	0.7169	0.9865
0.3529	0.7778	0.5	1	0.8059	5.3894
0	1	13.9598	20.6044	0.1942	0.0487
0.3186	1.0295	729.905	354.6389	0.7708	0.7273
0.0686	1.0197			0.8059	5.3498
0.0042	0.0458			3.5042	5.0971
0.0021	0.0295			0.6514	0.8453
0.0013	0.0268			0.0971	0.6143
0.0005	0.0011			4.4033	82.1862
0	1			0.0691	5.0061
14	9			−0.5785	−0.4245
0.5356	0.5571			2.307	3.2239
0.3072	0.501788				
0.1738	0.207122				

**Table 3:** Feature reduction based on rough sets.

Feature subset	Reduction results	Dimensionality
RS1	fs4, fs16, fs17, fs18, fi2, fi4, fi6, fi7, fi8, ft2, ft4, ft5, ft6, ft7, ft8, ft9, ft10, ft11, ft13, ft14, ft15, ft16	21
RS2	fs4, fs9, fs16, fs18, fi1, fi2, fi5, ft2, ft5, ft6, ft8, ft9, ft10, ft11, ft12, ft13, ft15	17
RS3	fs9, fs17, fs18, fi1, fi2, fi5, fi7, fi8, ft2, ft6, ft7, ft8, ft9, ft10, ft11, ft12, ft14, ft15, ft16	19
RS4	fs9, fs16, fs18, fi1, fi2, fi5, fi7, fi8, ft5, ft6, ft7, ft8, ft9, ft10, ft11, ft12, ft14, ft15, ft16	19
RS5	fs9, fs16, fs17, fs18, fi1, fi2, fi4, fi5, fi7, fi8, ft2, ft5, ft6, ft7, ft8, ft9, ft10, ft12, ft15, ft16	20

**Table 4:** Statistics of effectiveness before and after rough set reduction.

	Serial number	Accuracy (%)	Sensibility (%)	Specificity (%)	Processing time (s)
Before reduction	1	96.42	92.86	100	1.0610
	2	91.96	83.93	100	0.6170
	3	95.54	100	91.07	0.5490
	4	89.28	100	78.57	0.5630
	5	95.54	91.07	100	0.5470
	6	98.21	96.43	100	0.5460
	7	94.64	89.29	100	0.5460
	8	95.53	91.07	100	0.5460
	9	91.96	83.93	100	0.5460
	10	97.32	100	96.64	0.5300

	Mean	94.64	92.86	96.43	0.6051

After reduction (Rs1)	1	100	100	100	0.9370
	2	100	100	100	0.4360
	3	100	100	100	0.3870
	4	100	100	100	0.4210
	5	100	100	100	0.4210
	6	100	100	100	0.3900
	7	100	100	100	0.4060
	8	91.67	100	83.33	0.4060
	9	100	100	100	0.3740
	10	100	100	100	0.3930

	Mean	99.17	100	98.33	0.4571

Increase after reduction		4.53	7.14	1.9	0.148

**Table 5:** Effectiveness of rough set reduction subsets.

Subset	Average accuracy (%)	Average sensitivity (%)	Average specificity (%)	Processing time (s)
RS1	99.17	100	98.33	0.4571
RS2	97.5	96.67	98.33	0.4650
RS3	99.17	100	98.33	0.4656
RS4	100	100	100	0.4731
RS5	98.33	98.33	98.33	0.4850

Mean	98.83	99	98.66	0.4672

**Table 6:** Stability statistics of rough set reduction subsets.

	Training set/testing set	Accuracy (%)	Sensitivity (%)	Specificity (%)	Running time (s)
Before fusion	50/20	97.35	94.71	100	0.4873
	40/30	96.53	93.08	98.32	0.3846
	35/35	95.83	92.39	97.79	0.4254
	30/40	96.16	95.58	96.74	0.3560
	20/50	94.88	94.63	95.86	0.4236

	Mean	96.15	94.08	97.742	0.4154

After fusion (Rs1)	50/20	99.71	99.41	100	0.2684
	40/30	98.96	99.58	98.46	0.2568
	35/35	98.65	99.23	98.08	0.2382
	30/40	98.37	98.60	98.14	0.2646
	20/50	98.25	97.67	98.84	0.2636

	Mean	98.79	98.84	98.70	0.2583

**Table 7:** Classification performance of rough set reduction subset.

Subset	Average accuracy (%)	Average sensitivity (%)	Average specificity (%)	Running time (s)
RS1	99.17	100	98.33	0.2583
RS2	97.5	96.67	98.33	0.2870
RS3	99.17	100	98.33	0.2560
RS4	100	100	100	0.2531
RS5	98.33	98.33	98.33	0.2656

Mean	98.834	99	98.66	0.2620

**Table 8:** Classification performance of feature reduction based on PCA.

Serial number	Accuracy (%)	Sensitivity (%)	Specificity (%)	10 × running time (s)
1	91.67	83.33	100	0.9970
2	96.74	93.48	100	0.4830
3	96.74	93.48	100	0.4880
4	98.91	100	97.83	0.4950
5	93.48	86.96	100	0.4950
6	96.74	100	93.48	0.5140
7	96.74	100	93.48	0.5120
8	94.57	89.13	100	0.4890
9	97.83	95.65	100	0.4990
10	95.65	93.48	97.83	0.5180

Mean	95.91	93.55	98.26	0.5490

**Table 9:** Comparison of the performance of different lung nodule detection methods.

Author	Database	Nodule numbers	Accuracy (%)	FP/s
Santos et al. [11]	L	260	88.4	1.17
Magalhães Barros Netto et al. [12]	L	48	90.65	0.138
Ye et al. [13]	Pr	220	90.2	8.2
Tan et al. [14]	L	172	87.5	4
Cascio et al. [16]	L	148	97	6.1
Our method	Pr	70	99.17	0.47
